# A diagnostic framework to identify vestibular involvement in multi‐sensory neurological disease

**DOI:** 10.1111/ene.16216

**Published:** 2024-01-21

**Authors:** Amanda J. Male, Sarah L. Holmes, Nehzat Koohi, Magdalena Dudziec, Michael G. Hanna, Gita M. Ramdharry, Chiara Pizzamiglio, Robert D. S. Pitceathly, Diego Kaski

**Affiliations:** ^1^ SENSE Research Unit, Department of Clinical and Movement Neurosciences, Institute of Neurology University College London London UK; ^2^ NHS Highly Specialised Service for Rare Mitochondrial Disorders, Queen Square Centre for Neuromuscular Diseases The National Hospital for Neurology and Neurosurgery London UK; ^3^ Department of Neuromuscular Diseases UCL Queen Square Institute of Neurology London UK; ^4^ Queen Square Centre for Neuromuscular Diseases The National Hospital for Neurology and Neurosurgery London UK

**Keywords:** diagnosis, dizziness, neurotology, vertigo

## Abstract

**Background and purpose:**

Identifying vestibular causes of dizziness and unsteadiness in multi‐sensory neurological disease can be challenging, with problems typically attributed to central or peripheral nerve involvement. Acknowledging vestibular dysfunction as part of the presentation provides an opportunity to access targeted vestibular rehabilitation, for which extensive evidence exists. A diagnostic framework was developed and validated to detect vestibular dysfunction, benign paroxysmal positional vertigo or vestibular migraine. The specificity and sensitivity of the diagnostic framework was tested in patients with primary mitochondrial disease.

**Methods:**

Adults with a confirmed diagnosis of primary mitochondrial disease were consented, between September 2020 and February 2022. Participants with and without dizziness or unsteadiness underwent remote physiotherapy assessment and had in‐person detailed neuro‐otological assessment. The six framework question responses were compared against objective neuro‐otological assessment or medical notes. The output was binary, with sensitivity and specificity calculated.

**Results:**

Seventy‐four adults completed the study: age range 20–81 years (mean 48 years, ±SD 15.05 years); ratio 2:1 female to male. The framework identified a vestibular diagnosis in 35 participants, with seven having two diagnoses. The framework was able to identify vestibular diagnoses in adults with primary mitochondrial disease, with a moderate (40–59) to very high (90–100) sensitivity and positive predictive value, and moderate to high (60–74) to very high (90–100) specificity and negative predictive value.

**Conclusions:**

Overall, the clinical framework identified common vestibular diagnoses with a moderate to very high specificity and sensitivity. This presents an opportunity for patients to access effective treatment in a timely manner, to reduce falls and improve quality of life.

## INTRODUCTION

Dizziness and unsteadiness are frequent in people with neurological disease, as part of multi‐sensory dysfunction. Primary mitochondrial diseases (PMDs) are genetic conditions caused by pathogenic variants in genes involved in oxidative phosphorylation. Despite huge heterogeneity within PMDs, cells with high energy demands including nerves, eyes and ears are frequently affected [1]. PMDs therefore represent a model from which multi‐sensory impairment for other clinical presentations can be investigated [2].

People with PMDs very often report dizziness and unsteadiness, frequently attributed to cerebellar or peripheral nerve involvement (ataxia or neuropathy) [2, 3]. However, a high prevalence of vestibular dysfunction was reported within a heterogeneous cohort of people with PMD that included peripheral and central vestibulo‐cerebellar dysfunction, vestibular migraine (VM) and benign paroxysmal positional vertigo (BPPV) [3]. Established evidence of the benefits of targeted vestibular rehabilitation [4] introduces a unique opportunity for treatment to improve symptoms and function for people with PMD, for whom limited interventions exist.

Identification of vestibulopathy in PMD can be challenging given the presence of other sensory and motor impairments that affect balance. A diagnostic framework was developed and validated to identify vestibular causes of dizziness and unsteadiness (Table S1). This was then tested in multi‐sensory neurological disease (Figure S1) [5]. Here, the specificity and sensitivity of this framework is explored as a practical tool to identify common causes of vestibular dysfunction in people with multi‐sensory neurological disease.

## METHODS

**FIGURE 1:**
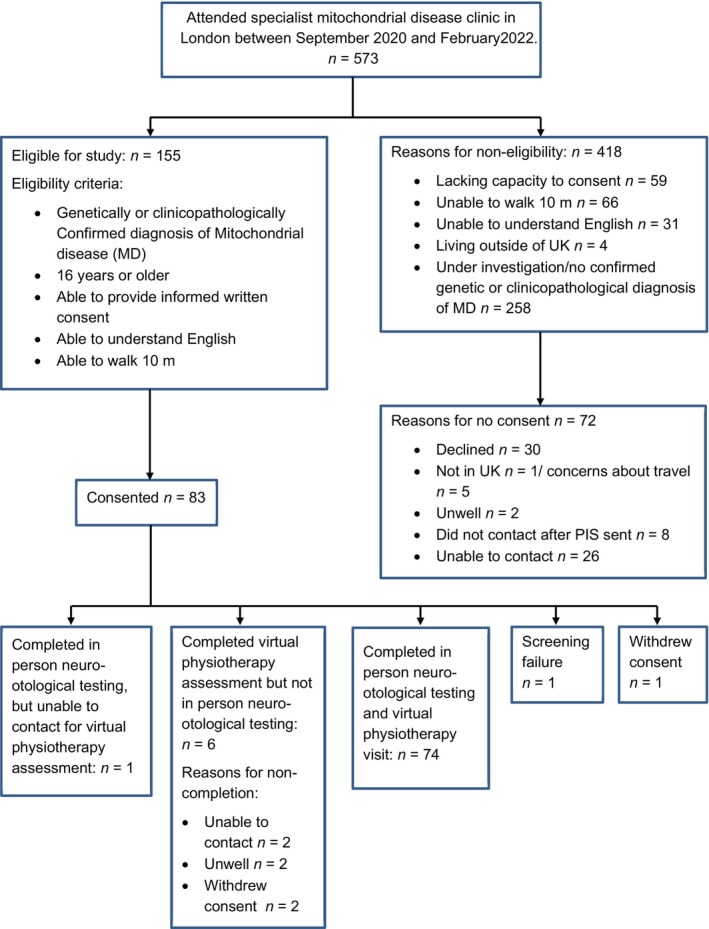
CONSORT diagram to outline the eligibility criteria and participant flow through the study.

Adults with PMDs (16 years of age or over) attending the specialist mitochondrial disease clinic in London were invited to participate in this prospective observational study (see Figure 1). Adults with and without dizziness or unsteadiness were included. Ethical approval was obtained (20/YH/0014). A sample size of 100 was identified to estimate anticipated sensitivity of 80% (including a margin of error of ±16%) and specificity of 90% for identifying vestibular dysfunction (including a margin of error of ±17%) with 95% confidence intervals.

A research physiotherapist completed remote physiotherapy assessment, including subjective screening of muscle strength and sensation. A research audiologist (with 18 years’ experience in vestibular testing) then completed detailed in‐person neuro‐otological assessment (Table S2) in a clinic setting, or at home for those identified as potentially being at high risk of more serious illness due to the COVID‐19 pandemic [6]. Framework questions (Figure S1) to identify VM, BPPV and vestibular dysfunction were also asked at this time.

Descriptive statistical analyses were performed on continuous and categorical data. Categorical data are presented as frequencies with percentages; continuous data are presented as means and standard deviations (±SD) where data are normally distributed. Medians and interquartile ranges are used when the data are not normally distributed.

As there are no diagnostic tests for VM, medical notes were used to collect information on the presence/absence of this. Medical notes for those with VM identified by the framework and neuro‐otological testing were interpreted by a blinded neurologist with expertise in neuro‐otology (DK).

The number of vestibular diagnoses identified by the framework (index test), namely VM, BPPV and vestibular dysfunction, were tested for accuracy against neuro‐otology testing and medical notes (reference standards). To reflect diagnostic methodology in clinical practice, data were divided into two subgroups: those compared to neuro‐otological tests, specifically vestibular dysfunction and BPPV; those compared to medical notes, namely VM. Accuracy of the diagnostic framework was prospectively assessed, using qualitative descriptors for sensitivity, specificity, positive predictive value and negative predictive values (NPV) [7].

## RESULTS

Seventy‐four adults were recruited, consented and completed virtual physiotherapy assessment and neuro‐otology testing (see Figure 1). Age range was 20–81 years (mean 48 years, ±SD 15.05 years); 2:1 female to male ratio. The most prevalent diagnosis was m.3243A>G *MT‐TL1* (57%). Remaining participants were diagnosed with other mitochondrial DNA mutations (24%), nuclear mutations (16%), m.3243A>T *MT‐TL1* (1%) and a clinicopathological diagnosis (1%). The median Newcastle Mitochondrial Disease Adult Scale score was 13, with an interquartile range of 11.75 (6.25–18). See Table S3 for details on diagnosis and multisystem characteristics affecting balance for all recruited participants.

Of the cohort, the framework identified vestibular dysfunction in 23% (17/75), BPPV in 1% (1/75) and VM in 13% (10/75). Two diagnoses were identified in 9% (7/75), specifically VM and vestibular dysfunction in 8% (6/75) and vestibular dysfunction and BPPV in 1% (1/75). The framework did not identify vestibular diagnoses in 53% (40/75).

Neuro‐otology testing confirmed vestibular dysfunction in 35% (26/75) and cerebellar dysfunction in 1% (1/75). VM was identified from medical notes in 12% (9/75). No vestibular diagnosis was detected in 47% (35/75). Two concurrent diagnoses were confirmed in 5% (4/75), VM and PVD in 3% (2/75), VM and BPPV in 1% (1/75) and vestibular dysfunction and BPPV in 1% (1/75).

Table 1 summarizes the results of the analysis for sensitivity and specificity.

**TABLE 1:** Overall results for sub‐categories.

	Framework identifying VD and/or BPPV, compared to neuro‐otological tests	Framework identifying BPPV, compared to neuro‐otological tests	Framework identifying VD, compared to neuro‐otological tests	Framework identifying VM compared to documentation in medical notes
Sensitivity				
Value (95% CI)	0.56 (0.37, 0.73)	1.00 (1.00, 1.00)	0.48 (0.30, 0.66)	0.50 (0.24, 0.76)
Descriptor	Moderate	Very high	Moderate	Moderate
Specificity				
Value (95% CI)	0.73 (0.57, 0.89)	1.00 (1.00, 1.00)	0.73 (0.57, 0.89)	0.87 (0.69, 1.05)
Descriptor	Moderate to high	Very high	Moderate to high	High
PPV				
Value (95% CI)	0.62 (0.44, 0.79)	1.00 (1.00, 1.00)	0.58 (0.40, 0.76)	0.44 (0.18, 0.70)
Descriptor	Moderate to high	Very high	Moderate	Moderate
NPV				
Value (95% CI)	0.68 (0.50, 0.85)	1.00 (1.00, 1.00)	0.64 (0.47, 0.82)	0.89 (0.73, 1.06)
Descriptor	Moderate to high	Very high	Moderate to high	High

*Note*: Qualitative descriptors for sensitivity, specificity, positive predictive power and negative predictive power values: <10 very low; 10–24 low; 25–39 low to moderate; 40–59 moderate; 60–74 moderate to high; 75–89 high; 90–100 very high [7].

Abbreviations: BPPV, benign paroxysmal positional vertigo; CI, confidence interval; NPV, negative predictive value; PPV, positive predictive value; VD, vestibular dysfunction; VM, vestibular migraine.

## DISCUSSION

A diagnostic framework was developed and validated to detect vestibular dysfunction, BPPV or VM in a vestibular neurology clinic. This was then tested for accuracy in a large cohort of patients with PMDs. Overall, the framework was able to identify vestibular diagnoses in adults with PMD, with a moderate (40–59) to very high (90–100) sensitivity and positive predictive value, and moderate to high (60–74) to very high (90–100) specificity and NPV [7].

The framework had moderate to high levels of specificity and NPV in ruling out vestibular dysfunction and/or BPPV, compared to neuro‐otological tests. Therefore, the framework offers a quick and simple tool that could be used in non‐specialist care settings to rule out these two vestibular diagnoses without the need for onward referral. Furthermore, the framework had moderate ability to correctly identify a diagnosis of vestibular dysfunction and/or BPPV, compared to neuro‐otological tests. This provides a unique opportunity to identify these diagnoses with good levels of confidence and guide onward referrals to specialist neuro‐otological or vestibular physiotherapy services to access evidence‐based treatment options [4].

Benign paroxysmal positional vertigo is reported to be the commonest cause of vertigo worldwide and thus prevalent in patients with other neurological disorders. Three questions were included in the framework to identify BPPV. Compared to neuro‐otological positional (Dix−Hallpike and roll) tests, these three questions had 100% accuracy at ruling in and ruling out a diagnosis of BPPV. Whilst only two participants were identified with BPPV in the study period, these three questions could help clinicians who do not have the expertise to complete the positional manoeuvres, or are managing patients virtually, to rule out BPPV with confidence. Those identified as having BPPV by these framework questions could then be referred to specialists for treatment.

Despite only having one question for detecting vestibular dysfunction [5], this observational study indicates that the diagnostic framework could rule out vestibular dysfunction at a moderate to high level of accuracy compared to neuro‐otological tests. The framework also had moderate levels of accuracy and confidence to identify a diagnosis of vestibular dysfunction. Identifying vestibular dysfunction in a group where dizziness and unsteadiness are frequently attributed to ataxia and neuropathy will enable patients to access vestibular rehabilitation.

In this study video head impulse testing was used as an objective measure of vestibular dysfunction, interpreting low vestibulo‐ocular gain as a feature of peripheral vestibular dysfunction and the presence of high velocity saccades following the head impulse as a feature of central vestibulopathy, where gains were normal [8]. Regardless of the origin of the vestibular dysfunction, both peripheral and central vestibular dysfunction can benefit from vestibular rehabilitation [4, 9]; hence this is why in this study it was chosen not to pursue a differentiation between peripheral and central aetiologies—a difficult separation in patients with PMDs where both peripheral and central vestibular pathways can be affected [10].

The diagnostic framework had high levels of specificity and NPV to rule out a diagnosis of VM compared with medical notes and could therefore prevent unnecessary use of preventative migraine medications. This is particularly relevant for a disorder such as VM where there are no diagnostic tests and a possible rate of over‐diagnosis amongst neurologists. The framework had moderate levels of confidence to rule in a diagnosis of VM, and this may reflect clinical practice whereby a targeted history guides diagnosis, rather than objective tests.

Limitations of this study include comparing the diagnosis of VM from the framework against medical notes. However, conducting standardized interviews of every participant was not feasible for this study. Additionally, there were only small numbers of patients who presented with BPPV. Therefore, larger scale validation for BPPV is recommended. Finally, the desired sample size was not attained, given high risk PMD participants needing to isolate during the COVID‐19 pandemic. Despite this, the framework was able to identify vestibular dysfunction, BPPV or vestibular migraine with moderate to very high levels of accuracy and to rule out these diagnoses at a moderate to high to very high level of accuracy, key priorities of a screening tool [7].

To conclude, a simple clinical diagnostic framework was developed and validated for identifying vestibular dysfunction, BPPV or VM, in patients with PMD, as an example of multi‐sensory neurological disease. The six framework questions are quick to complete, when patients report dizziness or unsteadiness. Larger scale validation studies will be required to assess its effectiveness in non‐specialist clinical settings.

## AUTHOR CONTRIBUTIONS

**Amanda J. Male:** Investigation; funding acquisition; writing – original draft; conceptualization; methodology; writing – review and editing; project administration; formal analysis. **Sarah L. Holmes:** Conceptualization; investigation; funding acquisition; writing – original draft; writing – review and editing; methodology; project administration; formal analysis. **Nehzat Koohi:** Writing – review and editing; investigation. **Magdalena G. Dudziec:** Investigation; writing – review and editing; methodology; data curation. **Michael G. Hanna:** Writing – review and editing. **Gita M. Ramdharry:** Conceptualization; formal analysis; supervision; writing – review and editing; methodology. **Chiara Pizzamiglio:** Writing – review and editing; data curation. **Robert D. S. Pitceathly:** Conceptualization; methodology; funding acquisition; writing – review and editing; supervision. **Diego Kaski:** Conceptualization; methodology; supervision; writing – review and editing; funding acquisition; investigation.

## FUNDING INFORMATION

The clinical and diagnostic ‘Rare Mitochondrial Disorders’ Service in London is funded by the UK NHS Highly Specialised Commissioners. The University College London Hospitals/University College London Queen Square Institute of Neurology sequencing facility receives a proportion of funding from the Department of Health's National Institute for Health Research Biomedical Research Centres funding scheme. A.J.M., S.L.H., M.G.H., G.M.R., C.P., R.D.S.P., and D.K. received funding from The Lily Foundation. N.K. and M.D. roles were funded by The Lily Foundation grant. R.D.S.P. is supported by a seedcorn award from the Rosetrees Trust and Stoneygate Foundation. N.K. is supported by a grant from the National Institute for Health and Care Research (HEE/NIHR ICA Programme Clinical Lectureship NIHR302201). C.P. is supported by the Clore Duffield Foundation. R.D.S.P. is supported by a Medical Research Council (UK) Clinician Scientist Fellowship (MR/S002065/1). R.D.S.P. and M.G.H. are supported by a Medical Research Council award (MC_PC_21046) to establish a National Mouse Genetics Network Mitochondria Cluster (MitoCluster) and by a Medical Research Council strategic award (MR/S005021/1) to establish an International Centre for Genomic Medicine in Neuromuscular Diseases (ICGNMD). S.L.H. and D.K. are supported by the National Institute for Health and Care Research, University College London Hospitals Biomedical Research Centre.

## CONFLICT OF INTEREST STATEMENT

The authors declare that they have no conflict of interest.

## Supporting information


Figure S1

Table S1

Table S2

Table S3

## Data Availability

The data that support the findings of this study are available from the corresponding author upon reasonable request.
