# Supplementary material for: A diagnostic framework to identify vestibular involvement in multi‐sensory neurological disease
Source: Eur J Neurol. 2024 Jan 21;31(5):e16216. doi: 10.1111/ene.16216 (PMC11235777; doi:10.1111/ene.16216)
Supplement: Supplementary file 1 — Figure S1 [file ENE-31-e16216-s002.docx]

**This diagnostic framework is designed to help identify vestibular involvement in multi-sensory neurological disease.**

**Patient reports:**

Dizziness, imbalance, light-headedness, unsteady gait, falls to the floor

Do you experience bouncing or blurred vision while walking?

***Yes □ No □***

***If yes …***

In at least 50% of the dizziness episodes, do you have:

- a headache with at least two of the following:-
  - one sided location
  - pulsating quality
  - moderate or severe pain intensity
  - or aggravation by routine physical activity?

***Yes □ No □***

- light (photophobia) or sound sensitivity (phonophobia)?

***Yes □ No □***

***If yes to both questions…***

Do you have:

- Dizziness when laying down in bed?

***Yes □ No □***

- Dizziness turning over in bed?

***Yes □ No □***

- Dizziness that lasts a few seconds as opposed to it being continuous?

***Yes □ No □***

***If yes to two questions…***

Vestibular dysfunction

Vestibular Migraine

BPPV

**Recommended management:**

Refer for Neuro-Otology investigations

Refer for vestibular rehabilitation

Manage migraine with lifestyle advice, acute and prophylaxis medication (as indicated)

Refer for vestibular rehabilitation (as required)

Complete positional tests and repositioning manoeuvres

Refer for vestibular rehabilitation (as required)

**Specific additional questions to ask:**
