# Supplementary material for: A diagnostic framework to identify vestibular involvement in multi‐sensory neurological disease
Source: Eur J Neurol. 2024 Jan 21;31(5):e16216. doi: 10.1111/ene.16216 (PMC11235777; doi:10.1111/ene.16216)
Supplement: Supplementary file 2 — Table S1 [file ENE-31-e16216-s004.docx]

|  | | Framework identifying VD and/or BPPV, compared to neuro-otological tests | Framework identifying BPPV, compared to neuro-otological tests | Framework identifying VD, compared to neuro-otological tests | Framework identifying VM compared to documentation in medical notes |
| --- | --- | --- | --- | --- | --- |
| Sensitivity | Value (95%CI) | 0.89 (0.74, 1.03) | 0.83 (0.5, 1.13) | 0.91 (0.74, 1.08) | 0.91 (0.74, 1.08) |
|  | Descriptor | High | High | Very high | Very high |
| Specificity | Value (95%CI) | 1.00 (1.00, 1.00) | 1.00 (1.00, 1.00) | 0.97 (0.86, 1.07) | 1.00 (1.00, 1.00) |
|  | Descriptor | Very high | Very high | Very high | Very high |
| PPV | Value (95%CI) | 1.00 (1.00, 1.00) | 1.00 (1.00, 1.00) | 0.91 (0.74, 1.08) | 1.00 (1.00, 1.00) |
|  | Descriptor | Very high | Very high | Very high | Very high |
| NPV | Value (95%CI) | 0.88 (0.72, 1.03) | 0.97 (0.84, 1.10) | 0.97 (0.86, 1.07) | 0.97 (0.87, 1.07) |
|  | Descriptor | High | Very high | Very high | Very high |

***Supplementary Information Table 1 – Overall results for framework sub categories for the validation cohort of 40 patients attending a tertiary outpatient vestibular neurology clinic.***
VD – vestibular dysfunction, BPPV – benign paroxysmal positional vertigo, VM – vestibular migraine, PPV – positive predictive value, NPV – negative predictive value, 95%CI – 95% confidence interval.

Qualitative descriptors for sensitivity, specificity, positive predictive power, and negative predictive power values: <10 Very low; 10–24 Low; 25–39 Low-moderate; 40–59 Moderate; 60–74 Moderate-high; 75–89 High; 90–100 Very high^[7]^

Forty consecutive patients attending a tertiary outpatient vestibular neurology clinic completed tested the framework: age range 17-81years (mean 54.7years, ±SD 15.5years); ratio 2:1 female to male. The framework identified a vestibular diagnosis in 24 participants, with two having two diagnoses.
