# Supplementary material for: A diagnostic framework to identify vestibular involvement in multi‐sensory neurological disease
Source: Eur J Neurol. 2024 Jan 21;31(5):e16216. doi: 10.1111/ene.16216 (PMC11235777; doi:10.1111/ene.16216)
Supplement: Supplementary file 3 — Table S2 [file ENE-31-e16216-s003.docx]

| Item | | Details |
| --- | --- | --- |
| Vestibular system | Videonystagmography (VNG) | An oculomotor examination is completed whilst wearing video goggles to record and analyse the eye movements.  Central and peripheral eye movement abnormalities can be detected. |
|  | Video Head Impulse Test (vHIT) | This tests the vestibular ocular reflex and can be completed for all three semi-circular canals of each ear.  The person is seated looking at a dot on the wall, while their head is moved quickly a small amount in the plane of each canal. The person wears goggles so any eye movements can be detected that indicate peripheral and central vestibular dysfunction. |
|  | Cervical Vestibular Evoked Myogenic Potentials (cVEMP) | A pressure wave is delivered to the inner ear (through a sound), whilst the evoked myogenic potential in the sternocleidomastoid muscle belly on the same side is recorded. This test measures the function of the saccule and the inferior portion of the vestibular nerve . |
|  | Dix Hallpike and Roll tests (positional tests) | These tests include watching for nystagmus whilst the person is laid down with their head supported in a certain position. Eye movements are recorded using video goggles. The presence of nystagmus in these tests typically indicates BPPV. |
| Visual dependence | Rod and disc test with virtual reality | A person views a screen through virtual reality goggles that block peripheral vision and is asked to adjust the position of a rod using a handheld controller until it is perceived as vertical, while the surrounding background moves. |

***Supplementary Information Table 2 – Details of the vestibular tests completed by each participant.***

Additional balance-related questionnaires and hearing assessments were carried out during the visit as part of a larger study (manuscript in preparation).
