# Supplementary material for: A diagnostic framework to identify vestibular involvement in multi‐sensory neurological disease
Source: Eur J Neurol. 2024 Jan 21;31(5):e16216. doi: 10.1111/ene.16216 (PMC11235777; doi:10.1111/ene.16216)
Supplement: Supplementary file 4 — Table S3 [file ENE-31-e16216-s001.docx]

| DIAGNOSIS | | | | | | | | | | | | | | Vestibular symptoms reported? | MULTISYSTEM FACTORS AFFECTING BALANCE AND DIZZINESS | | | | | | | | | | FRAMEWORK DIAGNOSIS | NEURO-OTOLOGY DIAGNOSIS |
| --- | --- | --- | --- | --- | --- | --- | --- | --- | --- | --- | --- | --- | --- | --- | --- | --- | --- | --- | --- | --- | --- | --- | --- | --- | --- | --- |
| GENETIC DIAGNOSIS | MUTANT LOAD (%) | | | | Sum completed NMDAS (25) | PHENOTYPE | | | | | | | |  | Migraine | Previous Stroke | Ataxia | Hearing | Vision | SENSATION (foot and ankle) | | | Muscle Strength | Fatigue |  |  |
|  | Age-adjusted blood m.3243A>G heteroplasmy | Blood | Urine | Muscle |  | MIDD | MERRF | MELAS | SANDO | Kearns-Sayre syndrome | Leigh syndrome | Asymptomatic | Other |  |  |  |  |  |  | Temperature | Pain | Light touch |  |  |  |  |
| m.3243A>G, *MT-TL1* | 38 | 8 |  | 78 | 14 |  |  |  |  |  |  |  | Y | Y |  |  | Y | bSNHL | NL | √ | √ | - | NL | Y | VM | VD |
| m.3243A>G, *MT-TL1* | 28 | 5 | 28 |  | 3 |  |  |  |  |  |  |  |  |  |  |  |  | bSNHL | RP | √ | √ | √ | NL |  | Nil | Nil |
| m.3243A>G, *MT-TL1* | 56 | 9 |  |  | 22 | Y |  |  |  |  |  |  |  | Y |  |  |  | bSNHL | R, M | √ | √ | √ | NL |  | VD | VD |
| m.3243A>G, *MT-TL1* | 70 | 23 |  |  | 39 |  |  | Y |  |  |  |  |  | Y | Y |  |  | bSNHL | R | - | x | - | - |  | Nil | VD |
| m.3243A>G, *MT-TL1* | 60 | 14 | 48 | 80 | 33 | Y |  |  |  |  |  |  | Y | Y |  |  |  | bSNHL | CRVO | √ | √ | x | - |  | VM & VD | VD |
| m.3243A>G, *MT-TL1* | 87 | 23 | 71 | 85 | 11 |  |  | Y |  |  |  |  |  | Y |  |  |  | bSNHL | NL | √ | √ | √ | NL |  | VD | VD |
| m.3243A>G, *MT-TL1* | 34 | 12 | 31 |  | 35 |  |  |  |  |  |  |  | Y | Y |  |  | Y | bSNHL | NL | √ | √ | - (L) | - |  | Nil | Nil |
| m.3243A>G, *MT-TL1* | 71 | 21 | 14 |  | 21 |  |  |  |  |  |  |  | Y | Y | Y | Y |  | bSNHL | R | √ | √ | - | - |  | VM & VD | VD |
| m.3243A>G, *MT-TL1* |  |  |  |  | 17 |  |  |  |  |  |  |  | Y | Y | Y |  |  | bSNHL | NL | √ | √ | √ | NL | Y | BPPV & VD | BPPV & VD |
| m.3243A>G, *MT-TL1* | 57 | 20 | 54 |  | 11 | Y |  |  |  |  |  |  |  |  |  |  |  | bSNHL | NL | √ | √ | √ | NL |  | Nil | Nil |
| m.3243A>G, *MT-TL1* | 8 | 2 | 11 |  | 3 |  |  |  |  |  |  |  |  |  |  |  |  | NL | NL | √ | √ | √ | NL |  | Nil | Nil |
| m.3243A>G, *MT-TL1* |  |  | 45 |  | 6 |  |  |  |  |  |  |  |  |  |  |  |  | bSNHL | NL | √ | √ | √ | NL | Y | Nil | VD |
| m.3243A>G, *MT-TL1* | 96 | 22 |  |  | 3 | Y |  |  |  |  |  |  |  |  |  |  |  | uCI & uSNHL | NL | - | - | - | NL |  | Nil | Nil |
| m.3243A>G, *MT-TL1* | 43 | 14 |  |  | 5 |  |  |  |  |  |  | Y |  |  | Y |  |  | NL | NL | √ | √ | √ | NL |  | Nil | Nil |
| m.3243A>G, *MT-TL1* | 61 | 20 |  |  | 2 |  |  |  |  |  |  |  |  |  |  |  |  | bSNHL | NL | √ | √ | √ | NL |  | Nil | Nil |
| m.3243A>G, *MT-TL1* | 65 | 17 |  |  | 18 | Y |  |  |  |  |  |  |  | Y |  |  |  | bSNHL | NL | √ | √ | √ | NL |  | Nil | VD & VM |
| m.3243A>G, *MT-TL1* | 43 | 10 | 62 |  | 6 |  |  |  |  |  |  |  |  | Y |  |  |  | bSNHL | R | √ | √ | √ | NL | Y | VD | VD |
| m.3243A>G, *MT-TL1* | 35 | 7 | 32 |  | 25 |  |  |  |  |  |  |  | Y | Y | Y |  | Y | bSNHL | R | √ | √ | - (L) | - |  | Nil | VD |
| m.3243A>G, *MT-TL1* | 91 | 28 |  |  | 5 |  |  |  |  |  |  |  |  |  |  |  |  | bSNHL | NL | √ | √ | √ | NL |  | Nil | VD |
| m.3243A>G, *MT-TL1* | 48 | 9 | 40 |  | 16 | Y |  |  |  |  |  |  |  | Y |  |  |  | bSNHL | NL | √ | √ | √ | NL |  | VD | VD |
| m.3243A>G, *MT-TL1* | 32 | 9 |  |  | 13 | Y |  |  |  |  |  |  |  | Y |  |  |  | bSNHL | NL | √ | √ | √ | NL |  | VD | VD |
| m.3243A>G, *MT-TL1* | 35 | 5 |  |  | 23 | Y |  |  |  |  |  |  |  | Y |  |  |  | bSNHL | DR | √ | - | - | - |  | VD | Nil |
| m.3243A>G, *MT-TL1* | 11 | 3 |  |  | 15 | Y |  |  |  |  |  |  |  | Y |  |  |  | NL | NL | √ | √ | - (L) | NL |  | VM | VM |
| m.3243A>G, *MT-TL1* | 100 | 37 |  |  | 18 | Y |  |  |  |  |  |  |  | Y |  |  |  | bSNHL | NL | √ | √ | - | NL |  | Nil | Nil |
| m.3243A>G, *MT-TL1* | 73 | 19 |  |  | 15 | Y |  |  |  |  |  |  | Y | Y |  |  |  | bSNHL | NL | √ | √ | √ | NL |  | Nil | VD |
| m.3243A>G, *MT-TL1* | 57 | 14 |  |  | 12 | Y |  |  |  |  |  |  |  | Y |  | Y |  | bSNHL | M | √ | √ | √ | NL |  | Nil | VD |
| m.3243A>G, *MT-TL1* | 100 | 59 |  |  | 2 |  |  |  |  |  |  | Y |  |  |  |  |  | NL | NL | √ | √ | √ | NL |  | Nil | Nil |
| m.3243A>G, *MT-TL1* |  |  | 52 |  | 15 |  |  |  |  |  |  |  | Y | Y | Y |  |  | bSNHL | NL | √ | - | - (L) | NL |  | VD | VD |
| m.3243A>G, *MT-TL1* | 67 | 29 |  |  | 3 |  |  |  |  |  |  | Y |  |  | Y |  |  | NL | NL | √ | √ | √ | NL |  | VM | Nil |
| m.3243A>G, *MT-TL1* | 69 | 31 | 71 |  | 5 |  |  |  |  |  |  |  | Y |  |  |  |  | NL | NL | √ | √ | √ | NL | Y | VD | Nil |
| m.3243A>G, *MT-TL1* | 50 | 17 | 48 |  | 5 |  |  |  |  |  |  |  |  | Y |  |  | Y | bSNHL | NL | √ | √ | √ | NL | Y | Nil | VD |
| m.3243A>G, *MT-TL1* | 91 | 38 |  |  | 13 |  |  |  |  |  |  |  | Y |  | Y |  |  | bSNHL | M | √ | √ | - | NL |  | VM | Nil |
| m.3243A>G, *MT-TL1* | 80 | 31 | 51 |  | 0 |  |  |  |  |  |  | Y |  |  |  |  |  | bSNHL | NL | √ | √ | √ | NL |  | Nil | Nil |
| m.3243A>G, *MT-TL1* | 51 | 17 |  |  | 9 |  |  |  |  |  |  |  |  | Y | Y |  |  | bSNHL | RP | √ | √ | - | NL |  | VM | Nil |
| m.3243A>G, *MT-TL1* | 73 | 11 |  |  | 15 | Y |  |  |  |  |  |  |  | Y |  |  |  | bSNHL | NL | √ | √ | √ | NL |  | VD | VD |
| m.3243A>G, *MT-TL1* | 77 |  | 46 |  | 10 |  |  |  |  |  |  |  |  |  | Y |  |  | NL | NL | √ | √ | √ | NL |  | VM | VD & VM |
| m.3243A>G, *MT-TL1* | 100 | 31 | 34 |  | 15 | Y |  |  |  |  |  |  |  | Y | Y |  |  | bSNHL | VI | √ | √ | - | NL |  | VM & VD | VD |
| m.3243A>G, *MT-TL1* | 100 | 66 | 73 |  | 3 |  |  |  |  |  |  |  | Y |  | Y |  |  | uSNHL | NL | √ | - | √ | NL |  | Nil | Nil |
| m.3243A>G, *MT-TL1* | 74 | 22 | 49 |  | 6 | Y |  |  |  |  |  |  |  |  |  |  |  | uSNHL | NL | √ | √ | - | NL |  | Withdrew from study | |
| m.3243A>G, *MT-TL1* | 48 | 10 |  |  | 20 | Y |  |  |  |  |  |  |  | Y | Y | Y |  | bSNHL | M | √ | √ | √ | NL |  | VM & VD | VM |
| m.3243A>G, *MT-TL1* | 4 | 2 |  |  | 4 |  |  |  |  |  |  |  |  |  | Y |  |  | NL | NL | √ | √ | √ | NL | Y | Nil | Nil |
| m.3243A>G, *MT-TL1* |  |  |  | 59 | 7 |  |  |  |  |  |  |  |  |  |  |  |  | bSNHL | NL | √ | √ | x | NL | Y | Nil | Nil |
| m.3243A>G, *MT-TL1* |  | 62 |  |  | 10 |  |  |  |  |  |  |  | Y |  |  | Y |  | bSNHL | NL | √ | √ | √ | NL | Y | Nil | VD |
| m.3243A>G, *MT-TL1* |  |  | 86 |  | 30 | Y |  |  |  |  |  |  |  |  | UTC |  |  | bSNHL | CPEO, M | UTC | UTC | UTC | UTC |  | VD | VD |
| m.3243A>T, *MT-TL1* |  |  |  | 75 | 17 |  |  |  |  |  |  |  | Y | Y | Y |  |  | NL | CPEO | √ | √ | √ | NL |  | VM | Nil |
| m.8344A>G, *MT-TK* |  |  | 70 |  | 19 |  | Y |  |  |  |  |  |  | Y | Y |  |  | bSNHL | OA | - (L) | - (L) | - | - |  | VD | VD |
| m.8344A>G, *MT-TK* |  | 53 |  |  | 7 |  | Y |  |  |  |  |  |  | Y |  |  |  | bSNHL | NL | √ | √ | - | - |  | Nil | VD |
| m.8344A>G, *MT-TK* |  | 86 |  |  | 27 |  | Y |  |  |  |  |  |  | Y |  |  |  | bSNHL | NL | √ | √ | - | NL |  | Nil | Nil |
| m.8344A>G, *MT-TK* |  | 58 |  |  | 3 |  | Y |  |  |  |  |  |  |  |  |  |  | uSNHL | NL | √ | √ | √ | NL |  | Nil | Nil |
| m.8344A>G, *MT-TK* |  | 79 |  |  | 21 |  | Y |  |  |  |  |  |  | Y |  |  |  | bSNHL | NL | - (L) | √ | √ | - |  | VD | VM |
| m.10158t>c, *MT-ND3* |  | 2 | 38 | 39 | 12 |  |  |  |  |  |  |  |  | Y |  |  |  | bSNHL | NL | √ | √ | - | - | Y | VD | Nil |
| m.6579G>A, *MT-CO1* |  | 39 |  | 68 | 24 |  |  | Y |  |  |  |  |  |  | Y | Y |  | bSNHL | NL | √ | - (L) | x (L) - (R) | - |  | Withdrew from study | |
| m.12258A>G, *MT-TS2* |  |  | 78 | 30 | 13 |  |  |  |  |  |  |  |  | Y |  |  | Y | bSNHL | RP | √ | √ | - | NL |  | VD | Nil |
| m.13513G>A, *MT-ND5* |  | 2 | 19 |  | 5 |  |  |  |  |  |  |  |  |  | Y |  |  | NL | NL | √ | √ | - | NL | Y | Nil | VM |
| m.14674T>C, *MT-TE* |  |  |  | 100 | 7 |  |  |  |  |  |  |  | Y |  |  |  |  | NL | NL | - | √ | x | NL | Y | Withdrew from study | |
| m.14674T>C, *MT-TE* |  |  |  | 100 | 26 |  |  |  |  |  |  |  | Y |  |  |  |  | bSNHL | NL | √ | √ | √ | NL |  | Nil | Nil |
| m.4300A>G, *MT-TI* |  | 100 |  |  | 0 |  |  |  |  |  |  | Y |  |  | Y |  |  | NL | NL | √ | √ | √ | NL |  | VD | Nil |
| m.10038G>A, *MT-TG* |  | 15 | 40 | 92 | 14 |  |  |  |  |  |  |  |  | Y | Y |  |  | bSNHL | RP | √ | √ | √ | NL | Y | Nil | VD |
| mtDNA rearrangement |  |  |  |  | 10 |  |  |  |  | Y |  |  |  | Y |  |  |  | NL | CPEO | √ | √ | √ | NL |  | Nil | VM |
| Multiple mtDNA deletions |  |  |  |  | 9 |  |  |  |  |  |  |  |  |  |  |  |  | uCI & uSNHL | CPEO | √ | √ | √ | NL |  | Nil | Nil |
| Single mtDNA deletion |  |  |  |  | 8 |  |  |  |  |  |  |  |  |  |  |  |  | NL | CPEO | √ | √ | √ | NL |  | Nil | Nil |
| Single mtDNA deletion |  |  |  |  | 10 |  |  |  |  |  |  |  | Y |  |  |  |  | NL | CPEO | √ | √ | √ | - |  | Nil | Nil |
| Single mtDNA deletion |  |  |  |  | 8 |  |  |  |  |  |  |  | Y |  |  |  |  | NL | CPEO | √ | √ | - (L) | - |  | Nil | Nil |
| Single mtDNA deletion |  |  |  |  | 14 |  |  |  |  |  |  |  | Y | Y | Y |  | Y | uSNHL | CPEO | √ | √ | √ | - |  | VM | VM |
| Single mtDNA deletion |  |  |  |  | 13 |  |  |  |  |  |  |  |  | Y |  |  |  | NL | CPEO | √ | √ | √ | NL |  | Nil | VD |
| Single mtDNA deletion |  |  |  |  | 9 |  |  |  |  |  |  |  |  |  |  |  |  | NL | CPEO | √ | √ | √ | NL |  | Nil | Nil |
| Multiple mtDNA deletions (nuclear maintenance gene panel negative) |  |  |  |  | 18 |  |  |  |  |  |  |  |  | Y | Y |  |  | bSNHL | CPEO | √ | √ | √ | NL |  | VM &VD | VM |
| mtDNA duplication |  |  |  |  | 8 | Y |  |  |  |  |  |  |  |  |  |  |  | bSNHL | NL | √ | √ | - (L) | NL |  | Withdrew from study | |
| AR POLG |  |  |  |  | 59 |  | Y |  |  |  |  |  |  |  | Y |  |  | NL | P, OA | UTC | UTC | UTC | UTC | Y | Withdrew from study | |
| AD POLG |  |  |  |  | 6 |  |  |  |  |  |  |  |  | Y | Y |  |  | NL | NL | √ | √ | √ | NL | Y | VM | VM |
| AD POLG |  |  |  |  | 37 |  |  |  | Y |  |  |  |  | Y |  |  |  | bSNHL | CPEO | √ | √ | - (L) | - |  | VD | VD |
| AD OPA1 |  |  |  |  | 29 |  |  |  |  |  |  |  |  |  |  |  |  | bSNHL | OA | √ | √ | - | - |  | Withdrew from study | |
| AD OPA1 |  |  |  |  | 9 |  |  |  |  |  |  |  |  |  | Y |  |  | NL | OA | √ | √ | x | - |  | Withdrew from study | |
| AD OPA1 |  |  |  |  | 14 |  |  |  |  |  |  |  |  |  | Y |  |  | bSNHL | OA | √ | √ | - (L) | NL |  | Nil | Nil |
| AD PEO1 |  |  |  |  | 20 |  |  |  |  |  |  |  | Y | Y |  |  | Y | uSNHL | CPEO | √ | √ | √ | - |  | BPPV | BPPV & VM |
| AR RNASEH1 |  |  |  |  | 28 |  |  |  |  |  |  |  | Y | Y |  |  | Y | bSNHL | CPEO | √ | √ | √ | NL |  | VD | Nil |
| AR SURF1 |  |  |  |  | 9 |  |  |  |  |  | Y |  |  | Y | Y | Y |  | NL | NL | √ | √ | √ | NL |  | VM & VD | CD |
| AR SDH |  |  |  |  | 14 |  |  |  |  |  |  |  | Y |  |  |  |  | NL | NL | UTC | UTC | UTC | UTC | Y | Withdrew from study | |
| Clinicopathological diagnosis (mtDNA mutation excluded) |  |  |  |  | 14 |  |  |  |  |  |  |  | Y | Y |  |  |  | NL | CPEO | √ | √ | √ | NL |  | Nil | Nil |
| Clinicopathological diagnosis |  |  |  |  | 10 |  |  |  |  |  |  |  | Y |  | Y |  |  | NL | CPEO | √ | √ | - | - | Y | Nil | Nil |
| Clinicopathological diagnosis |  |  |  |  | 23 |  |  |  |  |  |  |  |  |  |  |  | Y | NL | P | √ | √ | √ | - | Y | Nil | VD |
| Clinicopathological diagnosis |  |  |  |  | 25 |  |  |  |  |  |  |  |  | Y |  |  |  | bSNHL | P | √ | √ | √ | NL | Y | Nil | Nil |
| Clinicopathological diagnosis |  |  |  |  | 15 |  |  |  |  |  |  |  |  | Y | Y |  |  | NL | OA | √ | x | - (L) | NL | Y | VM | VM |

**Supplementary information table 3: Participant demographics with details of multisystem factors affecting dizziness and unsteadiness.**

KEY: M male, F female, NMDAS Newcastle mitochondrial disease adult scale, mtDNA mitochondrial DNA, AD autosomal dominant, AR autosomal recessive, MIDD Maternally inherited diabetes and deafness, MERRF Myoclonic epilepsy with ragged red fibres, MELAS Mitochondrial encephalomyopathy, lactic acidosis and stroke like episodes, SANDO Sensory ataxic neuropathy, dysarthria and opthalmoparesis, Y yes, √ intact, NL normal, x absent, - reduced, L left, R right, UTC unable to complete, bSNHL bilateral sensorineural hearing loss, uSNHL unilateral sensorineural hearing loss, uCI unilateral cochlear implant, R retinopathy, RP retinitis pigmentosa, CPEO chronic progressive external ophthalmoplegia, M maculopathy, P ptosis, OA optic atrophy, CRVO central retinal vein occlusion, DR diabetic retinopathy, VI visual impaired, VD vestibular dysfunction, VM vestibular migraine, BPPV benign paroxysmal positional vertigo, CD cerebellar dysfunction, Nil no vestibular diagnosis.
